# Autosomal Recessive Myotonia Congenita in an Adolescent Boy With Novel Mutation: A Case Report With Discussion on Management

**DOI:** 10.7759/cureus.53981

**Published:** 2024-02-10

**Authors:** Palash Das, Debasis Panigrahi

**Affiliations:** 1 Pediatric Medicine, Kalinga Institute of Medical Sciences, Bhubaneshwar, IND; 2 Pediatric Neurology, Jagannath Hospital, Bhubaneshwar, IND

**Keywords:** chloride channelopathy, clcn1 mutation, becker variant, mexiletine, myotonia congenita

## Abstract

Congenital myotonia represents a rare group of genetically inherited conditions. It can be either autosomal dominant (Thomsen) or autosomal recessive (Becker). It is characterized by muscular hypertrophy, proximal weakness, and myotonia, or impaired relaxation after contraction. These are due to mutations in the CLC1 gene.

A 14-year-old male child presented with complaints of gradually progressive weakness for five years. Weakness was more pronounced in the proximal muscle groups. The weakness worsened after rest and improved with activity. This led to absenteeism and affected his school performance. Clinical examination showed generalized muscular hypertrophy with pronounced hypertrophy of the calf muscles. A neurological examination showed significant myotonia and impaired relaxation after making a fist. The diagnosis of myotonia was confirmed by electromyography, which produced a dive-bomber sound on insertion. Next-generation sequencing revealed a homozygous eight-base pair insertion in exon 19 of the CLCN1 gene. This mutation has not been reported in the existing literature for myotonia congenita. The child was started on mexiletine and improved significantly. Presently, the patient is on regular medications and doing well on follow-up.

Though rare, congenital myotonia is an important cause of neuromuscular weakness. It can be easily diagnosed with a thorough clinical examination and routine testing for myotonia in all children with weakness. The treatment is relatively simple and can give the patient significant relief.

Myotonia can be easily diagnosed clinically, and pharmacotherapy and proper monitoring can remarkably improve patients’ quality of life.

## Introduction

Congenital myotonia is a rare genetic disorder characterized by muscle stiffness and delayed relaxation following voluntary muscle contractions. It belongs to a group of neuromuscular disorders known as channelopathies, which are caused by mutations in ion channel genes [1]. This case report presents the clinical features, diagnostic challenges, and management of congenital myotonia in a 14-year-old child. Congenital myotonia typically manifests in childhood or early adolescence, with symptoms ranging from mild muscle stiffness to severe impairment of movement. Affected individuals often experience difficulty initiating and terminating muscle contractions, resulting in prolonged muscle stiffness [2]. The disorder is caused by mutations in the genes encoding skeletal muscle voltage-gated chloride channels, leading to impaired chloride ion conductance and abnormal muscle membrane excitability.

## Case presentation

**Figure 1:**
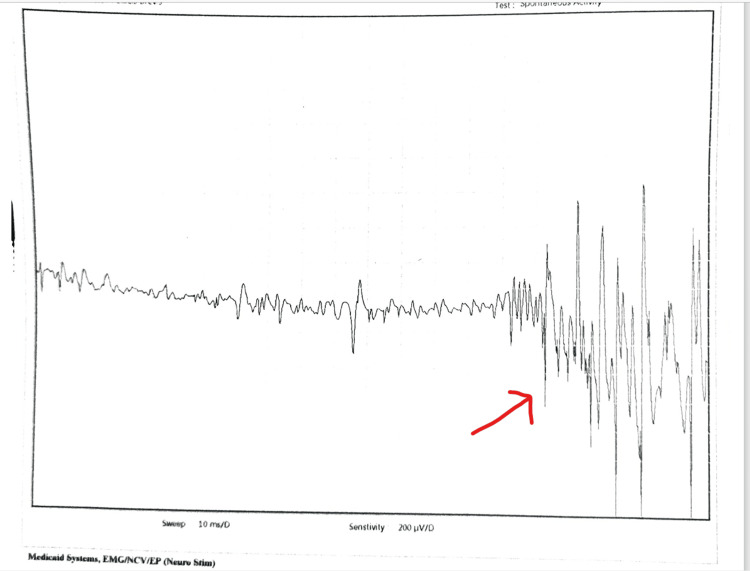
Electromyography showing myotonia

A 14-year-old male child presented with complaints of struggling to walk and get up from sitting. The parents said the child was doing well until age nine, when he started feeling tightness in his muscles; this tightness was more pronounced in the lower limbs, and he had progressive difficulty in ambulation. The weakness increased after a period of rest and improved with activity. There was no cognitive decline. He also developed calf muscle hypertrophy. He started to have three- to four-hour episodes of generalized muscle stiffness and difficulty moving and speaking before spontaneous recovery. These episodes became more frequent over the years, with stiffness that was persistent all day, easy fatigability, and difficulty walking. He usually recovered spontaneously with activity. He was born to third-degree consanguineously related parents with no family history of neuromuscular disorders. The perinatal and neonatal periods were uneventful. A general examination showed no dysmorphism, and there were no neurocutaneous markers. The neurological examination showed percussion-activated myotonia in the hands and legs. After a maximum voluntary contraction of the hand, he had marked difficulty opening his fist, but on repeated attempts, he improved because of the warm-up phenomenon. Investigations showed normal creatine phosphokinase levels. Electromyography (Figure 1) performed in the right abductor pollicis brevis muscle produced increased insertional activity and a dive-bomber sound on insertion and myotonic pattern. Motor unit potential showed decreased amplitude and activity.

The typical history of muscle stiffness, which worsened with activity, with clinical examination demonstrating calf hypertrophy and demonstrable myotonia on making a fist, a diagnosis of myotonia congenita was suspected. Relevant investigations were sent creatine phosphokinase (CPK) levels were 66.4 u/L (normal 24-195U/L). Electromyography was advised, and the findings suggested a diagnosis of myotonia congenita. For confirmation, whole-exome sequencing was sent.

Genetic testing showed a homozygous eight-base pair insertion in exon 19 of the CLCN1 gene (chr7: g.143346611_143346618dup; Depth: 113x) that results in a frameshift and premature truncation of the protein 22 amino acids downstream to codon 775 (p.Leu775PhefsTer22; ENST00000343257.7). This is a novel mutation not described earlier in the database (1000 genome, gnomAD, and Internal Database of the Medgenome). Both parents are carriers of this variant.

The child was started on the sodium channel blocker mexiletine. He was initially on 150 mg once daily and gradually increased to 450 mg/day. There was significant improvement, with a decrease in muscle stiffness and functional improvement in the child’s activity.

## Discussion

Myotonia congenita is a rare disorder and has been reported only from a few centers in India. The easy availability of next-generation sequencing has now identified many novel mutations that have not been described before [1]. Myotonia is clinically seen as impaired relaxation after voluntary contraction. Myotonia can be demonstrated in clinics by (a) asking patients to clench their fists for 10 seconds and then unclench their fists and (b) observing delayed and slow unclenching [1]. This exercise should be repeated 5-10 times to demonstrate an improvement in symptoms with repeated use (the warm-up phenomenon). Myotonia can also be demonstrated by percussing the thenar, forearm extensor, trapezius, quadriceps, and tongue muscles and by observing impaired relaxation after contraction.

Myotonia is caused by a chloride channel mutation, which is closely mimicked by para myotonia caused by a sodium channel mutation. Both mutations are associated with membrane hyperexcitability. However, repeated muscle use worsens para myotonia, and observing the warm-up phenomenon can help distinguish between the two diseases [1].

Genetically, two variants are reported: autosomal dominant (Thomsen) and autosomal recessive (Beckers). Chloride channels are primarily responsible for maintaining the sarcolemma membrane’s resting membrane potential. In the presence of reduced chloride conductance, there is a relative increase in potassium in the T tubules, which may account for self-sustained bursts of discharge that lead to myotonia [2].

Myotonia can be diagnosed with electromyography (EMG), which typically shows myotonic potential. Myotonic potentials are defined as repetitive discharges at 20-80 Hz [3]. The amplitude and frequency typically wax and wane. When played on audio, the sound has been described as resembling a dive-bomber aircraft or motorcycle engine. The confirmatory test is genetic sequencing of the CLC1 gene. More than 100 mutations have already been described, and many new mutations are being reported [2]. In our case, we found a mutation that has not been reported previously. Our patient had an onset in childhood, and it was an autosomal recessive variant [3] (Table 1).

**Table 1:** Difference between sodium channel and chloride channel mutation causing myotonia congenita EMG: Electromyography. Image credit: Debasis Panigrahi Author.

Clinical features	Sodium channel mutation	Chloride channel mutation
Myotonia	Present	Present
Response to cold	Usually worsens in response to cold	Some may be worsened by cold
Stiffness	More common in facial muscles	More common in lower limbs
Symptoms after repeated use	Para myotonia worsens after warm-up	Improves after warm-up
Pain	Common feature	Rarely seen
Treatment	May not respond well to mexiletine. Some may respond to acetazolamide.	Good response to mexiletine
EMG	Patterns I and III are seen	Pattern II is common

Congenital myotonia is typically treated with mexiletine [4]. Mexiletine is a class IB antiarrhythmic drug that works by blocking the sodium channel [5]. It increases the resting membrane potential (RMP) and reduces the hyperexcitability of the sarcolemma. Other medications, mainly lamotrigine and carbamazepine, can also be tried in the absence of mexiletine [5].

Current clinical management of non-dystrophic myotonia (NDM) includes muscle warming routines, specialist physiotherapy, and the avoidance of triggers. However, the clinical experts stated that pharmacological management should be offered to any person with NDM who is seeking treatment because it is affecting their daily lives. Sodium channel blockers (such as mexiletine, carbamazepine, acetazolamide, flecainide, and phenytoin) have been used off-label for many years to treat NDM. Off-label imported mexiletine was used most because the benefits can be seen very quickly. The clinical experts explained that a recent randomized controlled trial showed evidence of the efficacy of another sodium channel blocker, lamotrigine, which is also sometimes used if mexiletine is contraindicated, not effective, or not tolerated. The committee concluded that mexiletine is currently the preferred, established treatment for NDM, and other options are available when mexiletine is not suitable [6].

The patient’s response to mexiletine is often dramatic, and there is a reduction in myotonia and a remarkable reduction in fatiguability [1,7-8]. While counseling these patients, they must be made aware of a small but significant risk of muscle stiffness when succinylcholine is used as an anesthetic agent. It is an autosomal recessive disorder, and both parents are carriers for the same. Parents have to be counseled about the inheritance of the same, and the 25% risk of having an affected progeny in every pregnancy and the 50% risk of having a carrier must be clearly explained. 

## Conclusions

Myotonia is not life-threatening, but the quality of life is affected. Myotonia congenita can be noticed in the clinic with a strong clinical suspicion, followed by simple maneuvers like asking the patient to clench and unclench their fist. The diagnosis must be confirmed by electromyography, and genetic testing may be offered if available. All trials have shown a significant improvement in the quality of life of patients who have been treated on a long-term basis. In myotonia, pharmacotherapy with proper monitoring can remarkably improve patients’ quality of life. All patients deserve a correct diagnosis and should be adequately treated and receive follow-up for life. In our case, the child presented with clinical features consistent with myotonia, which were confirmed by findings in EMG studies. Since the child showed improvement after exercise, myotonia congenital was suspected. There is no element of progressive muscular weakness and wasting, cardiac disease, eye abnormalities, or endocrine disturbances in favor of myotonia congenita and not myotonic dystrophy. As the literature shows good results with mexiletine, though other sodium channel blockers like carbamazepine and phenytoin can be used, the child was started on it and showed clinical improvement. 
